# Circulating Neutrophils of Nonalcoholic Steatohepatitis Patients Show an Activated Phenotype and Suppress T Lymphocytes Activity

**DOI:** 10.1155/2020/4570219

**Published:** 2020-06-30

**Authors:** Laura Antonucci, Cristiana Porcu, Eleonora Timperi, Silvano Junior Santini, Gino Iannucci, Clara Balsano

**Affiliations:** ^1^Francesco Balsano Foundation, Rome, Italy; ^2^Dipartimento di Medicina Interna e Specialità Mediche, Sapienza Università di Roma, Rome, Italy; ^3^Department of Life, Health and Environment Sciences, University of L'Aquila, L'Aquila, Italy

## Abstract

Neutrophils or PolyMorphonuclear Neutrophils (PMNs) are key effector cells of the innate immune system and thanks to their remarkable plasticity, establish a cross talk with T cells modulating their survival and effector functions. During Nonalcoholic Steatohepatitis (NASH), the advanced form of hepatic steatosis or NAFL, PMNs infiltrate liver tissue, becoming a histological feature of NASH. Our aim was to evaluate the frequency of PMNs in NAFL and NASH patients in order to understand how they modulate the activity of circulating CD4^+^ and CD8^+^ T cells. In our cohort of patients, NASH patients displayed a higher frequency of circulating PMNs that was strongly correlated to liver enzymes, grade of steatosis, inflammation and fibrosis, the hepatocellular ballooning, and NAFLD Activity Score (NAS). Furthermore, even if *ex vivo*, in both groups of patients, PMNs shared the same phenotype of resting cells, after 24 hours of coculture with autologous CD4+ and CD8+ T cells, PMNs of NASH patients acquired a more active phenotype, becoming able to strongly inhibit proliferation and activation of CD4^+^ and CD8^+^ T cells. The higher ability of PMNs of NASH patients in suppressing CD4^+^ and CD8^+^ T cells, over time, might contribute in reducing the immunological defense of liver tissue against damages thus taking part in the progression of the NAFL disease toward NASH.

## 1. Introduction

Nonalcoholic fatty liver disease (NAFLD) is a very common chronic disease, ranging from simple hepatic steatosis (NAFL), due to an excessive fat deposition in the hepatocytes without any sign of inflammation or necrosis, to nonalcoholic steatohepatitis (NASH), featured by steatosis and hepatic inflammation [1–3]. NASH can lead to advanced fibrosis and cirrhosis, thereby increasing the risk of developing hepatocellular carcinoma (HCC) [4].

Despite NAFL affects around one-third of the Western world with an incidence that continues to grow, no pharmacological drugs have been already approved for the treatment of the disease. Currently, only lifestyle change (diet and physical activity) is suggested to these patients [5, 6].

During NASH disease, stressed or dying hepatocytes release intracellular molecules called damage-associated molecular patterns (DAMPs), such as high mobility group box 1 (HMGB1), that induce immune cells to initiate a homeostatic wound-healing response to repair liver injury [7]. Accordingly, DAMPs stimulate Kupffer cells (KCs) to produce guidance signals such as macrophage inflammatory protein-2 (MIP-2), interleukin-1*β* (IL-1 *β*), tumor necrosis factor-*α* (TNF-*α*), and interleukin-8 (IL-8) to recruit polymorphonuclear neutrophils (PMNs) within the portal tract [8, 9]. Amongst the others, IL-8 represents the most potent PMN chemo-attractants in inflammation, which, through binding with two different chemokine receptors C-X-C chemokine receptor type 1 (CXCR-1) and C-X-C chemokine receptor type 2 (CXCR-2), induces specific intracellular signaling cascades for a rapid PMNs recruitment [10, 11].

PMNs accumulation in the liver tissue is one of the main features of NASH, contributing to liver damage and exacerbating inflammatory state [12]. PMNs represent the most abundant circulating white blood cells in humans [13]. Their mobilization out of blood vessels into injured tissue is a highly regulated process that depends on sequential interactions between adhesion molecules present in the environment, such as selectins [L-selectin (CD62L), P-selectin (CD62P), E-selectin (CD62E)], integrins [integrin *α*M (CD11b), *β*2 integrin (*α*L*β*2)], and chemokines [14]. In particular, CD62L is expressed on PMNs surface and has an important role in the initial rolling and attachment of PMNs to the endothelium [15]. This is followed by tight adhesion and transendothelial migration mediated by integrins, such as CD11b. Therefore, the activation of PMNs is associated to the shedding of CD62L and the upregulation of CD11b. Once captured and activated, adherent PMNs migrate through the vessel wall to infiltrate the tissue [16].

PMNs functions are mediated by the expression levels of myeloperoxidase (MPO), arginase-1 (ARG1), and reactive oxygen species (ROS), which reflect PMN activity, and their involvement in inducing cellular injury and fibrosis [8, 17]. Activated PMNs promote the recruitment of lymphocytes by the release of mediators of inflammation and chemokines [18–20]. As a result, resident and infiltrated T cells release cytokines that, acting on the endothelial cells of the liver sinusoids, lead to the production of IL-8 for a further recruitment of PMNs. This interplay between PMNs and T cells intensifies the state of inflammation and creates a link between the adaptive and the innate immune system [20]. PMNs establish a close interaction with T cells to promote activation or suppression of their effector functions, influencing, in this way, the state of different pathologies, such as infections, sepsis, and cancer [21–24]. For example, in HIV-1 infected patients, PMNs suppress T cell activity lading to an acceleration of the disease [22]. PMNs have also antitumor and protumor activities. Accordingly, during the early phase of lung tumor growth, PMNs tend to inhibit primary tumor growth by recruiting and activating T cells [25–27]. On the contrary, in the late stage of cancer, PMNs show an immunosuppressive phenotype that suppresses antitumor T-cell responses and promotes immune evasion [28]. Thus, as extensively reported in the literature, PMNs can positively or negatively modulate T-cell functions. PMNs can activate a variety of T-cell subsets, while, the inhibition of T-cells by immunosuppression of PMNs occurs through the release of ARG1, ROS, and proteases, or via PD-L1-dependent interaction [20, 22].

The modulation of T cell responses by PMNs has not yet been studied in NAFL and NASH patients. This interplay is extremely important since it might influence the state and progression of NAFLD disease. We analyzed the frequency and phenotype of PMNs in healthy donors (HD), NAFL, and NASH patients wondering how PMNs may drive and modulate the activity of T cells in this disease.

## 2. Material and Method

### 2.1. Patients and Biochemical Parameters

Observational study on 10 healthy donors and 30 consecutive patients with a diagnosis of NAFL/NASH performed by ultrasound and by excluding the following criteria: no history of current or past excessive alcohol drinking as defined by an average daily consumption of alcohol <30 g/day in men and<20 g/day in women; negative tests for the presence of hepatitis B surface antigen and antibody to hepatitis C virus; absence of history and findings consistent with cirrhosis and other chronic liver diseases [29]. Human studies were performed in accordance with the ethical guidelines of the 1975 Declaration of Helsinki and approved by the Ethical Committee of the University of L'Aquila, L'Aquila, Italy (Prot. No. 31869). Informed consent was obtained from all enrolled patients.

All subjects, at the time of enrolment, had a complete work-up including a clinical examination, anthropometric measurements, laboratory tests, and a liver ultrasonography scan. Blood sampling was performed in the morning after an overnight fast. Patient characteristics are listed in Table 1.

Study population underwent fasting blood sampling to assess blood glucose (FBG), total cholesterol, HDL-cholesterol, triglycerides, aspartate aminotransferase (AST), alanine aminotransferase (ALT), *γ*-glutamyl transpeptidase (*γ*-GT), alkaline phosphatase, nitrogen, and creatinine by standard laboratory methods. Low-density lipoprotein (LDL) cholesterol value was obtained using the Friedwald formula. The homeostasis model assessment of insulin resistance (HOMA-IR) was calculated as previously described [30]. Peripheral blood neutrophil-to-lymphocyte ratio (NLR) was calculated dividing the number of neutrophils by the number of lymphocytes in peripheral blood sample.

Evaluation Liver ultrasonography scanning was performed to assess the degree of steatosis. All ultrasonography were performed by the same operator who was unaware of the aims of the study and blinded to laboratory values using an EsaoteMedica apparatus equipped with a convex 3,5 MHz probe.

Steatosis was graded according to Matsuda and DeFronzo [31] on the basis of abnormally intense, high-level echoes arising from the hepatic parenchyma, liver-kidney difference in echo amplitude, echo penetration into the deep portion of the liver, and clarity of liver blood vessel structure.

### 2.2. Liver Histology

Only in NASH group patients it was possible to carry out biopsy, as indicated in the American Association for the Study of Liver Diseases (AASLD) guidelines [32–34].

The histological diagnosis was established using Hematoxylin & eosin (H&E) and Masson trichrome stains of formalin-fixed paraffin-embedded liver tissue. All biopsies were evaluated by a pathologist blinded to patient data according to the NAFLD Activity Score- (NAS-) scoring system described by Kleiner et al. [35] to detect the number of patients who met the histological criteria for nonalcoholic steatohepatitis (NASH). The histological features of liver tissues, commonly described in NASH, including steatosis, inflammation (portal and lobular), hepatocyte ballooning, and fibrosis were scored as summarized in Table 2.

### 2.3. Enzyme-Linked Immunosorbent Assay (ELISA)

IL-8 (Diaclone, Cat# 855.080.005, RRID:AB_596520, France), HMGB1 (Tecan Trading AG, Switzerland), myeloperoxidase (MPO) (Enzo Life Sciences, Inc.), and arginase 1 (ARG1) (Elabscince, USA) concentrations were measured in the serum of NAFL and NASH patients, with specific immunoassay kits, according to the manufacturer's instructions. Absorbance was read at 450 nm with a photometer (Multiskan, flow cytometry, TermoFisher). All samples were analyzed in duplicate in the same run.

### 2.4. Isolation of PBMCs and PMNs

PMNs and peripheral blood mononuclear cells (PBMCs) were isolated from heparinized venous blood from NAFL and NASH patients. Cells were isolated by density gradient centrifugation by Lympholyte (Cedarlane, Burlington, ON, Canada). The low-density fraction of PBMCs was removed and, after wash, collected in complete RPMI 1640 medium containing 10% FBS (HyClone GE Healthcare Life Sciences), 2 mM L-6-glutamine (Sigma-Aldrich), penicillin/streptomycin (EuroClone).

PMNs obtained from high-density fraction by sedimentation (30 minutes) of the red blood cells with 3% Dextran/HBSS without calcium and magnesium (Sigma-Aldrich) followed by lysis of the erythrocytes by the usage of Red Blood Cell Lysis Solution (RBC 10x, MiltenyBiotec). To check the purity of isolated PMNs, CD11b (Thermo Fisher Scientific Cat# 14-0112-82, RRID:AB_467108) and CD16 (Thermo Fisher Scientific Cat# 46-0168-41, RRID:AB_1834390) markers are analyzed by flow cytometry (FC). The purity of PMNs exceeded the 90%. PMNs viability was greater than 98%, as determined by Trypan blue (Sigma-Aldrich) exclusion. PMNs were suspended in HBSS (without Ca^2+^/Mg^2+^) and placed on ice until use.

### 2.5. Flow-Cytometry Analysis

The list of Antibodies (Abs) used is available on Table 3.

Flow cytometric analysis was performed on PMNs and PBMCs of healthy donors, NAFL, and NASH patients. Dead cells in all samples were excluded using Fixable Viability Dye eFluor 780 (eBioscience) and Zombie Aqua Fixable Viability Kit (Biolegend). Surface staining was performed by incubating the cells with selected Abs at 4°C for 20 min in PBS 2% FBS. BD LSR Fortessa cell analyzer (BD Biosciences) was used to acquire the samples and analyzed with FlowJo software, version 10.0.8r1 (Treestar) according to the guidelines for the use of flow cytometry and cell sorting in immunological studies [36]. FMO (fluorescence minus one) staining control was stained with all the antibodies used in the experiment except one which was used as control.

### 2.6. PBMCs Functional Assays

PBMCs were resuspended in PBS (CambrexBioScience) and labeled with 1 *μ*M of carboxyfluoresceindiacetate, succinimidyl ester (CFSE) (Life Technologies, Thermo Fisher Scientific, Grand Island, NY, USA) for 20 min at 37°C. After incubation, the cells were washed with complete RPMI 1640 to block the excess of CFSE. Then, PBMCs were cultured in flat-bottomed 96-well plates (Corning B.V. Life Sciences, Amsterdam, Netherlands) in complete RPMI medium and were stimulated with Dynabeads Human T-Activator CD3/CD28 (Thermo-Fisher Scientific) at a ratio of 1 : 1. PMNs were added to autologous PBMCs, and after 4 days of culture, PBMC proliferation and CFSE dilution were analyzed by flow cytomety on gated CD4^+^ and CD8^+^ T lymphocytes.

### 2.7. Analysis of Markers of PMN Activation

CD62L and CD11b, markers of activation of PMNs, were analyzed in freshly PMNs isolated by pheripharial blood and after 1 day of coculture with autologous PBMCs to evaluate their activation status. After isolation ex *vivo*, PMNs of NAFL and NASH patients were added to autologous PBMCs at ratio 2 : 1. After 24 h, the surface staining was performed by incubating the cells with selected Abs at 4°C for 20 min in PBS 2% FBS. Samples were acquired on the BD LSR Fortessa cell analyzer and analyzed with FlowJo software. CD62 L and CD11b antibodies used for flow cytometry analysis were reported in Table 3. FMO (fluorescence minus one) staining control was stained with all the antibodies used in the experiment except one which was used as control. Flow cytometry compensation beads (Thermo-fisher) were used as positive control of CD62L and CD11B staining on fresh PMNs. PMNs stimulated with Lipopolysaccharide (LPS) (1 ug/mL, from Escherichia coli O111:B4) were used as a positive control for the detection of CD62L and CD11b expression in coculture experiments.

### 2.8. Analysis of the Viability of PMNs and PBMCs

The apoptosis of PBMCs and PMNs was assessed by labeling cells with Fixable Viability Dye and Annexin V (Apoptosis detection it-eBioscience). The cells were stained with Fixable Viability Dye for 30 min at room temperature (RT) and washed twice with PBS. Cells were labelled with CD45, CD3, CD4, and CD8 antibodies for the identification of CD4+/CD8+ T cells, while CD16, CD62L, and CD11b for detecting PMNs. All antibodies used for flow cytometry analysis were summarized in Table 3. Subsequently, 1/500 Annexin-V diluted was added in appropriated binding buffer (provided by eBioscience kit) for 25 minutes at RT. After incubation, cells were washed in PBS and analyzed by flow cytometry.

### 2.9. Measurement of Intracellular ROS

2′,7′-dichlorodihydrofluorescein diacetate (H2DCF-DA) fluorescent probe (Thermo Fischer Scientific) was used to identify the intracellular production of ROS in PMNs and PBMCs cell populations. The cells were stained with Fixable Viability Dye for 30 min at RT then PMNs and PBMCs were labeled with cocktail containing H2DCF-DA probe (10 *μ*M) and the following antibodies: CD45, CD3, CD4, and CD8 antibodies for the identification of CD4+/CD8+ T cells, while CD16, CD62L, and CD11b for detecting PMNs. PMNs stimulated with Lipopolysaccharide (LPS) (1 *μ*g/mL, from Escherichia coli O111:B4) were used as positive control for ROS detection.

### 2.10. Statistical Analysis

2-tailed paired/unpaired Student *t* test was used to analyze in *vitro* data. 2-tailed Mann-Whitney *U*-test was applied to compare groups of *ex vivo* samples. Correlations were calculated by using Spearman analysis. In all tests, differences were considered statistically significant when *P* value was less than 0.05. Statistical analysis was performed with Prism software (version 6; GraphPad Software, La Jolla, California).

## 3. Results

### 3.1. Frequency of PMNs and Inflammatory Mediators in NASH Patients

**Figure 1:**
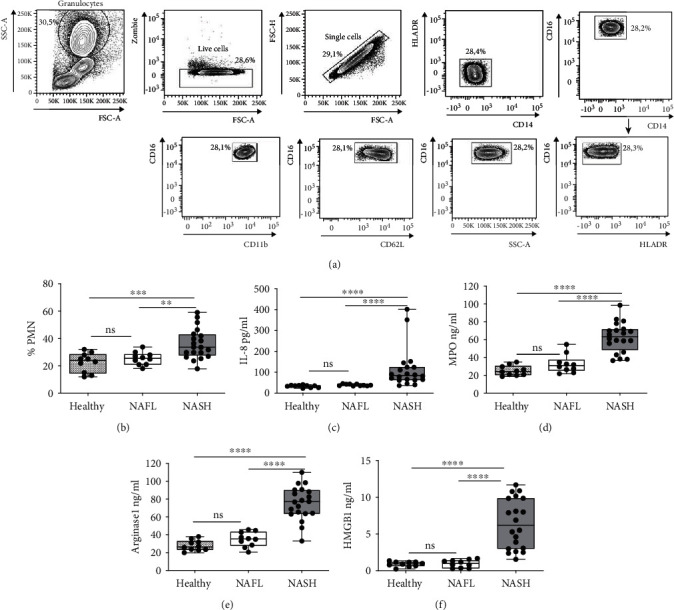
Frequency of PMNs and mediators of inflammation in Healthy Donors, NAFL, and NASH patients. (a) Representative gating strategy for the flow cytometric analysis of PMNs identification from peripharial blood. PMNs are identified as SSC^high^ CD16^high^ CD62L^high^ CD11b^high^. (b) Frequency was calculated by flow cytometry analysis as the percentage of CD16^high^ CD62L^high^ CD11b^high^ of PMNs in healthy donors (*n* = 10), NAFL (*n* = 10), and NASH patients (*n* = 20). Each dot in the dot-plot represents one subject/experiment. (c–f) Serum concentration of IL-8 pg/mL (Healthy *n* = 10, NAFL *n* = 10, and NASH *n* = 20) (c), MPO ng/mL (Healthy *n* = 10, NAFL *n* = 10, and NASH *n* = 20) (d), ARG1 ng/mL (Healthy *n* = 10, NAFL *n* = 10, and NASH *n* = 20) (e), and HMGB1 ng/mL (Healthy *n* = 10, NAFL *n* = 10, and NASH *n* = 20) (f) in healthy donors, NAFL, and NASH patients were quantified by ELISA analysis. Each dot in the graph represents a single subject. Each dot in the dot-plot represents one subject/experiment. Each subject has been studied in an independent experiment. Plots are representative for three independent experiment with one sample per experiment. Mann-Whitney unpaired test, 2-tailed. *P* value, ^∗^*P* < 0.05, ^∗∗^*P* < 0.01, ^∗∗∗^*P* < 0.001.

We performed an observational study on 10 healthy donors, 10 NAFL, and 20 NASH patients, enrolled consecutively by Predictive Medicine Center and Transfusion Center, Policlinico Umberto I-Hospitals-Rome Italy, respectively. PMN profile was examined in the peripheral blood (PB) of healthy donors, NAFL, and NASH patients; the gating strategy has been illustrated in Figure 1(a). We characterized PMNs based on CD16, CD62L, and CD11b expression by flow cytometric analysis. Monocytes and natural killer (NK) cells were excluded based on CD14 and HLA-DR positivity. No differences were observed in the percentage of classical, intermediate and nonclassical monocytes, and natural killer in the peripheral blood of healthy donors, NAFL, and NASH patients (Supplementary Figure S1). We estimated the percentage of PMNs, identified as SSC ^High^ CD16^High^ CD62L^High^ CD11b^High^, in PB of healthy donors, NAFL, and NASH patients.

We observed that the percentage of PMNs was comparable in NAFL patients and healthy donors, while it was significantly higher in NASH patients compared to healthy donors and NAFL patients (Figure 1(b)). IL-8, MPO, ARG1, and HMGB1 were analyzed in the serum of healthy donors and patients. Notably, all these proinflammatory factors were significantly increased in NASH compared to NAFL patients, while no difference was found between healthy donors and NAFL subjects (Figures 1(c)–1(f)).

### 3.2. PMN Frequency Directly Correlate to the Severity of the Liver Disease in NASH Patients

**Figure 2:**
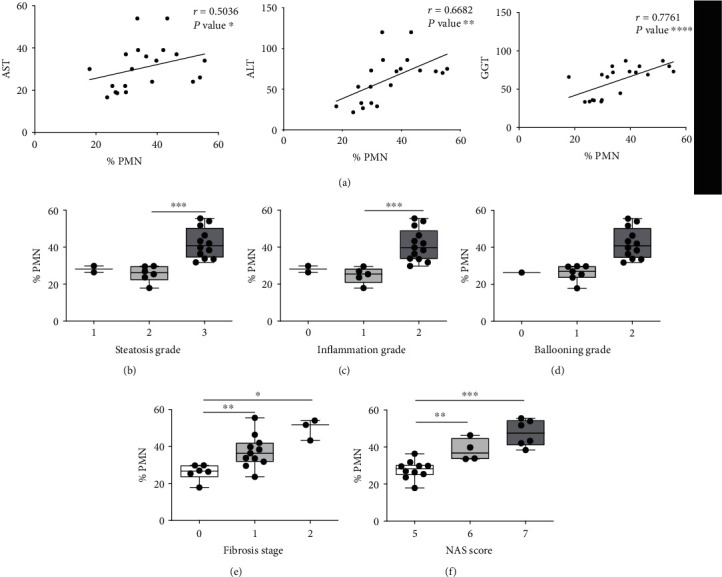
PMN frequency of NASH patients correlate with liver damage. (a) Spearman correlation (*r*) between the frequency of freshly PMNs isolated from peripherical blood of NASH patients (*n* = 20) and liver enzymes aspartate aminotransferase (AST), alanine aminotransferase (ALT), and *γ*-glutamyl transferase (GGT) in serum (*r* = 0.536, *r* = 6682, and *r* = 7761, respectively), data derived from medical records of patients. *P* value, ^∗^*P* < 0.05, ^∗∗^*P* < 0.01, ^∗∗∗^*P* < 0.001, and ^∗∗∗∗^*P* < 0.0001. (b–f) Boxplots represent the association between frequency calculated by flow cytometry analysis as a percentage of CD16^high^ CD62L^high^ CD11b^high^ of PMNs in NASH patients (*n* = 20) and histological severity of liver disease evaluated by steatosis grade (b), inflammation grade (c), hepatocellular ballooning (d), fibrosis stage (e), and NAS activity score (f). All data are represented as mean ± SD. Each dot in the box-plot represents one subject/experiment (*n* = 20). Each subject has been studied in an independent experiment. Plots are representative for three independent experiments with one sample per experiment. Mann-Whitney unpaired test, 2-tailed. *P* value, ^∗^*P* < 0.05, ^∗∗^*P* < 0.01, ^∗∗∗^*P* < 0.001, ∗∗∗∗*P* < 0.0001.

To evaluate the clinical implication of PMNs in the pathogenesis of NAFL/NASH liver disease, we correlated the frequency of PMNs, calculated by flow cytometry, with the biochemical parameters of patients. Notably, in NASH patients, we found a significantly positive correlation between PMN frequency and liver enzymes: AST (*r* = 0.5036; ^∗^*P* < 0.05), ALT (*r* = 0.6682; ^∗∗^*P* < 0.01), and GGT (*r* = 0.7761; ^∗∗∗∗^*P* < 0.0001) (Figure 2(a)). No correlations were observed between PMN frequency and alkaline phosphatase, total, and direct bilirubin (Supplementary Figure S2).

After that, in the liver tissue of NASH patients, the possible association between the percentage of PMNs and the severity of the liver damage was evaluated. We look at the grade of steatosis, inflammation and fibrosis, the hepatocellular ballooning, and NAFLD Activity Score (NAS) (Figures 2(b)–2(f), respectively). A strong association between the percentage of PMNs and the severity of liver damage was highlighted (Figures 2(b)–2(f)).

### 3.3. *Ex Vivo* PMNs Resting Phenotype in NAFL and NASH Patients

**Figure 3:**
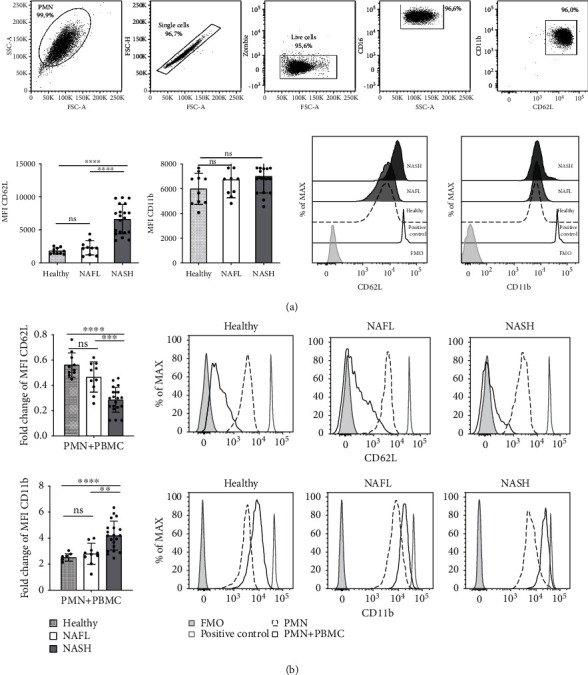
PMNs activated phenotype of NASH patients. (a) Top panels, representative flow cytometry gating strategy (NASH patients) for identification *ex vivo* PMNs. FACS plots displaying, SSC-A versus FSC-A, singlet cells (FSC-H versus FSC-A), live cells (Zombie versus FSC-A), CD16 versus SSC-A, CD11b versus CD62L in ex *vivo* PMNs analysis. Bottom-left: histogram of mean fluorescence intensity (MFI) of CD62L and CD11b of ex *vivo* PMNs flow cytometric analysis obtained from healthy donors (*n* = 10), NAFL (*n* = 10), and NASH patients (*n* = 20). Bottom-right: representative histogram of flow cytometry analysis with an overlay showing CD62L and CD11b MFI in gated CD16^High^CD62L^High^CD11b^High^ of healthy donors (dashed line), NAFL (light gray), and NASH patients (dark gray). FMO control was represented by a gray histogram. Flow cytometry compensation beads positive control was represented by gray line. (b) PMNs obtained from healthy donors, NAFL, and NASH patients after 24 h of coculture with (PMNs+PBMCs) or without PBMCs (PMNs). Left panels: fold changes of CD62L (top) and CD11b (bottom) of MFI gated in CD16^High^ CD62L^Dim^ CD11b^High^ in healthy donors (*n* = 10), NAFL (*n* = 10), and NASH patients (*n* = 20). Fold change was calculated comparing PMNs with PBMCs respect to PMNs without PBMCs. Right panels: representative histogram with an overlay showing CD62L (top) and CD11b (bottom) MFI in gated CD16^High^CD62L^High^CD11b^High^ after 24 h of coculture with PBMC in healthy donors, NAFL, and NASH patients. Histogram with dashed line represents PMN, PMNs+PBMCs are represented by a dark line. FMO control was represented by gray histogram and Lipopolysaccharide (LPS) positive control was represented by a gray line. In all histograms reported, for each subgroup, the distribution of each subject/experiment is shown, and results are expressed as mean ± SD. Each dot represents one subject/experiment. Each subject has been studied in an independent experiment. In all experiments, statistics were performed by Mann-Whitney unpaired test, 2-tailed. *P* value, ^∗∗^*P* < 0.01, ^∗∗∗^*P* < 0.001, ns: nonsignificant.

We evaluated CD62L and CD11b expression on circulating PMNs purified by healthy donors and NAFL and NASH patients. Ex vivo PMNs of healthy donors, NAFL, and NASH patients showed a resting cell phenotype: CD16^High^ CD62L^High^ CD11b^High^ (Figure 3(a)). Notably, PMNs of NASH patients showed a significant increase of baseline expression of CD62L respect to healthy donors and NAFL patients (Figure 3(a)). On the contrary, no significant difference in the expression of the CD11b was observed in our cohort of healthy donors and patients. CD62L and CD11b expression was estimated by Mean Fluorescence Intensity (MFI).

### 3.4. Strong Activation State and Prolonged Survival of PMNs in NASH Patients

After 24 hours of coculture with autologous PBMCs, we looked at the expression levels of CD62L and CD11b in PMNs of our cohort of patients (Figure 3(b)). Notably, PMNs of NASH patients displayed a significant decrease in CD62L expression levels and a significant increase in CD11b respect to healthy donors and NAFL patients (Figure 3(b), representative flow cytometry gating strategy showed in Supplementary Figure S3).

**Figure 4:**
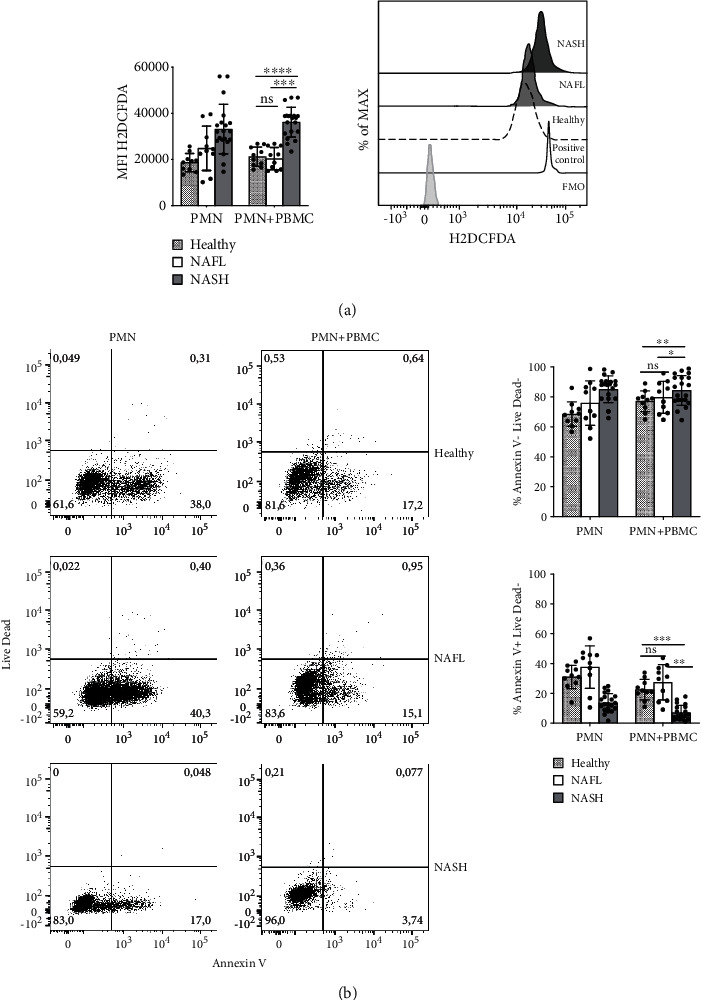
PMN ROS intracellular production and viability. (a) PMNs obtained from healthy donors, NAFL, and NASH patients after 24 h of coculture, with (PMNs+PBMCs) or without PBMCs (PMNs), were stained with H2DCF-DA probe for intracellular production of ROS and (b) Annexin-V/Live Dead for vitality. (a) Left panel: histogram of H2DCFDA (ROS) MFI of CD16^High^CD62L^High^CD11b^High^ cell subset obtained from healthy donors (*n* = 10), NAFL (*n* = 10), and NASH (*n* = 20). Right panel: representative histogram of flow cytometry analysis with an overlay showing H2DCFDA (ROS) MFI in gated CD16^High^CD62L^Dim^CD11b^High^ of healthy donors (dashed line), NAFL (light gray), and NASH patients (dark gray), after 24 h of coculture with PBMCs. FMO control is represented by gray histogram and LPS positive control by a gray line. (b) Left panel: representative plots of PMNs cell death of healthy donors, NAFL, and NASH patients, after 24 h, with (PMNS+PBMCs) or without PBMCs (PMNs). Right panel: percentage of live (Annexin-V, Live Dead negative) and early apoptotic (Annexin-V positive, Live Dead negative) of PMNs obtained from healthy donors (*n* = 10), NAFL (*n* = 10), and NASH patients (*n* = 20).In all histograms reported, for each subgroup, the distribution of each subject/experiment is showed and results are expressed as mean ± SD. Each dot represents one subject/experiment. Each subject has been studied in an independent experiment. In all experiments, statistics were performed by Mann-Whitney unpaired test, 2-tailed. *P* value, ^∗^*P* < 0.05, ^∗∗^*P* < 0.01, ^∗∗∗^*P* < 0.001, ^∗∗∗∗^*P* < 0.0001, ns: nonsignificant.

PMNs from NASH patients showed, at baseline, high levels of ROS production (Figure 4(a)). Comparing foldchange in ROS production, we highlighted a significant increase in ROS production in PMNs+PBMCs coculture (Supplementary Figure S4). PMNs of NASH patients showed a significant increase of cell survival (%Annexin V-/Live Dead-) respect to PMNs of HD and NAFL patients, regardless of the presence of PBMCs (Figure 4(b)). Accordingly, a significant decrease of cell death (%Annexin V+/Live Dead-) in PMN+PBMC of NASH patients respect to PMNs was observed (Figure 4(b)).

### 3.5. PMNs of NASH Patients Strongly Suppress CD4^+^ and CD8^+^ T Cells Proliferation and Activation

**Figure 5:**
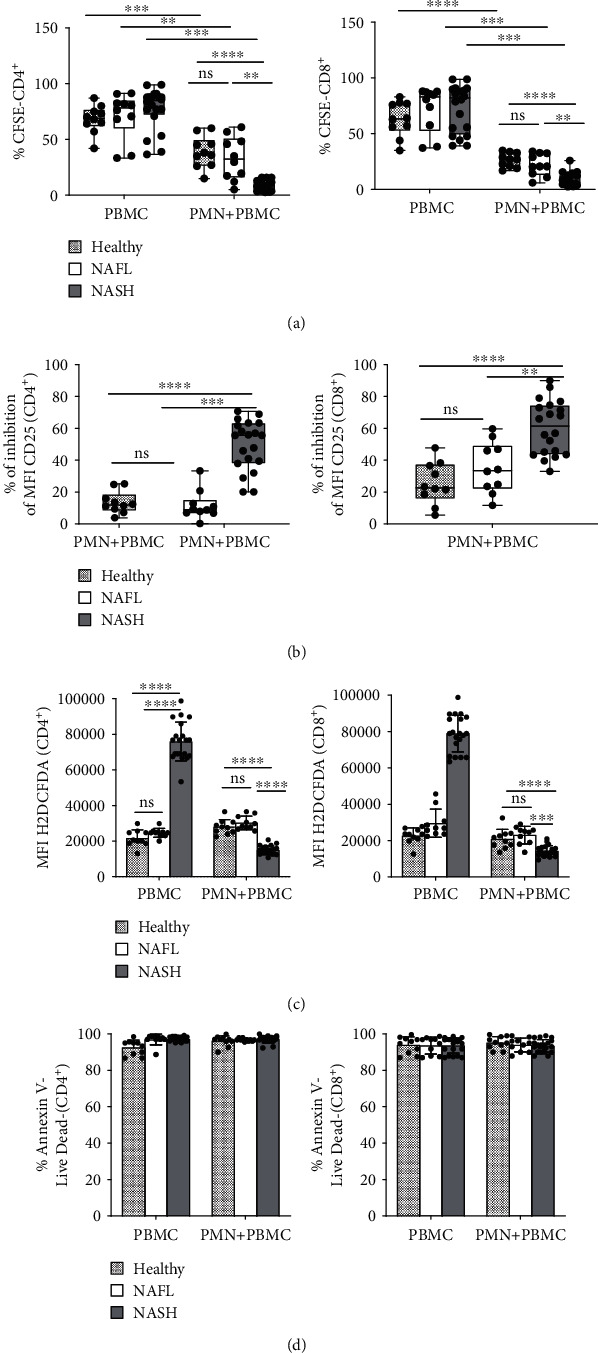
Proliferation and activation of CD4^+^ and CD8^+^ T cells cocultured with PMNs. (a–d) In all experiments, PBMCs obtained from healthy donors, NAFL, and NASH patients were stimulated with aCD3/aCD28 and cultured in the presence or in absence of autologous PMNs for 96 h. The capacity of PMN to suppress T cells was evaluated by measuring proliferating T cells (CFSE dilution). (a) Top panels: frequency calculated by flow cytometry analysis as the percentage of CD4^+^ (left) and CD8^+^ (right) T cell proliferation is measured in healthy donors (*n* = 10), NAFL (*n* = 10), and NASH (*n* = 20) by CFSE assay. Each dot in the graph represents a single subject. (b) The expression of activation marker (CD25) on T cells was analyzed by flow cytometry, after 96 h of cultured with PMNs. Dot plots show the percentage of inhibition of CD25 MFI [(MFI increase of CD25 of PBMC–MFI increase of CD25 of PBMC + PMN)/(MFI increase of CD25 of PBMC) x 100%] on CD4^+^ (left) and CD8^+^ (right) T cells in healthy donors (*n* = 10), NAFL (*n* = 10), and NASH (*n* = 20). Each dot in the dot-plot (a and b) represents one subject/experiment. Each subject has been studied in an independent experiment. Plots are representative for two independent experiment with one sample per experiment. (c–d) After 96 h of coculture with PMNs, T cells were stained with H2DCF-DA probe for identifying intracellular production of ROS, and Annexin-V/Live Dead for their vitality. (c) Histograms show the percentage of H2DCFDA (ROS) MFI on gated CD4^+^ (left) and CD8^+^ (right) T cells in healthy donors (*n* = 10), NAFL (*n* = 10), and NASH (*n* = 20). (d) Frequency of cell death CD4^+^ (left) and CD8^+^ (right) T cells in healthy donors (*n* = 10), NAFL (*n* = 10), and NASH (*n* = 20). Survival is defined as the percentage of Annexin V, Live Dead double negative cells. In all histograms reported (c and d), for each subgroup the distribution of each subject/experiment is showed and results are expressed as mean ± SD. Each dot represents one subject/experiment. Each subject has been studied in an independent experiment. In all experiments (a–d), statistics were performed by Mann-Whitney unpaired test, 2-tailed. *P* value, ^∗^*P* < 0.05, ^∗∗^*P* < 0.01, ∗∗∗*P* < 0.001, ∗∗∗∗*P* < 0.0001, ns: nonsignificant.

Since PMNs of NASH patients showed a more active phenotype, we looked at their capacity in affecting lymphocyte proliferation and activation. CFSE-based assay allowed us to assess the PMNs ability in modulating lymphocyte proliferation. PBMCs were isolated from healthy donors, NAFL, and NASH patients, labeled with proliferation tracer (CFSE) and stimulated with beads aCD3/aCD28, in the presence or absence of autologous PMNs. A representative gate strategy of CFSE labeled CD4^+^ and CD8^+^ T cells in the presence or not of autologous PMNs was shown in Supplementary Figure S5. After the CD3/aCD28 stimulation, both CD4^+^ and CD8^+^ T cells enhanced their proliferation in healthy donors and patients. Interestingly, the PMNs/PBMC coculture induced a significant decrease of CD4^+^ and CD8^+^ T cell proliferation only in NASH patients (Figure 5(a)).

To determine whether PMNs were able to modulate the activation of CD4^+^ and CD8^+^ T cells, we looked at the expression of the T cell activation marker, CD25. We also evaluated the ability of CD4^+^ and CD8^+^ T cells to produce ROS. CD4^+^ and CD8^+^ T cells of NASH patients, after coculture with autologous PMNs, showed a significant decrease of CD25 expression levels respect to healthy donors and NAFL patients (Figure 5(b)). We observed a higher baseline ROS production CD4+ and CD8+ T cells of NASH patients compared to healthy donors and NAFL patients (Figure 5(c)). On the contrary, accordingly with the downregulation of CD25, ROS production was significantly decreased in PMN+PBMC of NASH patients respect to healthy donors and NAFL patients (Figure 5(c)). This behavior was in line with the observed lack of apoptosis (Figure 5(d)).

## 4. Discussion

At the early stage, neutrophil infiltration in the liver represents one of the typical histological characteristics of NASH patients and animal models [37–39]. In response to chemotactic factors released by resident cells, KC, and T cells, PMNs migrate from the bloodstream to the liver through the sinusoidal endothelial fenestrae. PMN infiltration contributes to the progression of tissue damage through the release of proinflammatory mediators, such as MPO and ROS [17, 18, 37]. These factors are involved in lipid peroxidation and hepatic stellate cell migration, facilitating cellular injury and fibrosis. The key role of neutrophils in the pathogenesis of NASH diseases was supported by demonstrating the increase of circulating levels and activity of neutrophils elastase during the curse of this pathology [39, 40]. In NASH animal models, these proinflammatory cells regulate the ceramide metabolism, which is also involved in the pathogenesis of NASH [41].

Neutrophils show a strategic role not only in the early stage of NAFL/NASH disease but also in promoting the progression of NASH toward hepatocellular carcinoma (HCC) [42]. In particular, high levels of neutrophil extracellular traps (NETs) were found in the serum of patients with NASH, these clinical observations have been confirmed in mice models in which NET formation contributes to the progression of HCC [42].

Interestingly, in the last years, it has become increasingly evident that PMNs are engaged in complex bidirectional interaction with T cells, establishing correct environmental conditions to initiate, amplify, and/or suppress adaptive immune effector responses [20, 21]. Thus, PMNs play an important role in the regulation of T cell immune response, worsening or improving the state of inflammation and the progression of diseases [20, 37]. Nowadays, the crosstalk between PMNs and T lymphocytes, during NAFLD, has not been evaluated yet, but might have an important role in the progression of NAFL towards NASH. In fact, although it has been reported the highest number of PMNs in NASH patients [38, 43], an in-depth study of their complex interplay has not been already developed.

In our cohort of 30 NAFL/NASH patients, we observed a high Neutrophil-to-lymphocyte ratio (see Table 1) and a significant increase of PMNs frequency in the peripheral blood of NASH patients. In line with other studies [43], in the serum of our cohort of NASH patients, we observed an increase of IL-8, a neutrophil chemotactic factor. IL-8 might be involved in driving PMN mobilization towards the site of inflammation [18, 43]. In addition, the increased levels of HMGB1, ARG1, and MPO indicate a higher activation of PMNs, leaving to hypothesize the main role of these cells in NASH progression. The data reported above were also confirmed by both a positive correlation between the frequency of PMNs and AST, ALT, and GGT parameters, and the association of neutrophils activity with the severity of liver disease (Figures 2(a) and 2(b)).

Expression changes in cell adhesion molecules, such as CD62L and CD11b, correlate with increased chemotaxis, transendothelial migration, and leukocyte activation [29]. *Ex vivo* PMNs deriving from healthy donors, NAFL, and NASH patients showed a resting phenotype characterized by CD16^high^ CD62L^high^ and CD11b^high^ expression. Notably, we highlighted an overexpression of CD62L on the surface of PMNs of NASH patients respect to healthy donors and NAFL patients (Figure 3(a)). CD62L acts as a homing receptor and is required for the capture of circulating PMNs on activated endothelium [13, 14, 44–46]; thus, its overexpression could indicate a higher mobilization of PMNs towards the site of inflammation. No modulation was observed for CD11b in PMNs (Figure 3(a)). The lack of differences in the baseline expression levels of CD11b among all the three different groups of patients suggests a resting-state of circulating PMNs. Accordingly with literature, circulating PMNs of our cohort of patients seem to be not active because of their expression of low levels of CD11b that are associated with inactive conformation of ligand binding site of PMNs [47–49].

Interestingly, PMNs in coculture with autologous PBMCs showed an activated phenotype (CD16^High^ CD62L^Dim^ CD11b^High^). Notably, PMNs from NASH patients, conversely respect to healthy donors and NAFL, when cocultured with PBMCs, acquired a strong active phenotype as revealed by the shedding of CD62L, the upregulation of CD11b (Figure 3(b)) and the increased intracellular production of ROS [50] (Figure 4(a)). These evidences supported the idea that PMNs of NASH patients have an intrinsic capacity to be activated in the presence of PBMCs.

Moreover, PMNs of our cohort of NASH patients, in the presence of PBMCs, have longer survival and less apoptosis survival compared to PMNs of healthy donors and NAFL patients (Figure 4(b)). All these observations suggest that PMNs of NASH patients, in the presence of PBMCs, can contribute in worsening liver inflammatory status. The apoptosis observed in PMNs of healthy donors and NAFL patients could be a determinant for the effective resolution of liver inflammation.

Finally, for the first time, we have demonstrated that PMNs of NASH patients were able to suppress the proliferation and the activation of autologous CD4^+^ and CD8^+^ T cells compared to PMNs from healthy donors and NAFL patients (Figure 5(a)). This behavior is highlighted by the downregulation in CD4^+^and CD8^+^ T cells of the expression of CD25 (Figure 5(b)) together with the low intracellular concentration of ROS (Figure 5(c)) and lack of apoptosis (Figure 5(d)).

## 5. Conclusion

Taking all into consideration, our data revealed an extremely different behavior and function of PMNs in NASH respect to NAFL patients and healthy donors. These findings highlight that, in the presence of steatohepatitis, an immunological tolerance might take place; thus contributing to the progression of liver disease. Furthermore, a strong suppression of CD4^+^ and CD8^+^ T cell proliferation and activation may induce, over time, an inadequate immune-surveillance of liver damage, making patients more susceptible to liver damage progression.

The modulation of PMNs activity, in NASH patients, might represent a novel and effective therapeutic approach to counteract fat-related liver damage progression. Thus, the use of molecules able to modulate PMNs activity could redirect the immunosuppressive properties of PMNs and boost T-cell responses in NASH patients.

## Figures and Tables

**Table 1:** Anthropometric and biochemical parameters in healthy donors, NAFL, and NASH patients.

Parameters	Healthy donors (10)	NAFL (10)	NASH (20)	*P* value (NASH vs. healthy donors)	*P* value (NASH vs. NAFL)
Age (years)	54 ± 6.9	57.6 ± 7.2	56.3 ± 11.2	0.1682	0.8655
Sex (F/M)	5/5	4/6	12/8		
Weight (kg)	68.75 ± 5.3	69.4 ± 8.3	88.7 ± 17.3	<0.0001	<0.0001
BMI (kg/m^2^)	22.4 ± 1.68	23.0 ± 1.7	32.4 ± 4.7	<0.0001	<0.0001
AST (UI/mL)	11.2 ± 5.5	20.4 ± 6.5	30.7 ± 11.3	0.0046	0.0007
ALT (UI/mL)	12.1 ± 4.04	26.1 ± 5.53	62.8 ± 30.2	0.0006	0.0005
GGT (UI/mL)	18.52 ± 5.58	23.9 ± 7.39	61.2 ± 20.2	<0.0001	<0.0001
Total cholesterol (mg/dL)	161 ± 15.5	172.9 ± 24.1	212 ± 25.9	<0.0001	<0.0001
LDL cholesterol (mg/dL)	100.6 ± 20.9	109.7 ± 33.8	125 ± 19.5	0.0432	0.0339
HDL cholesterol (mg/dL)	48.0 ± 8.1	47.6 ± 7.3	46.8 ± 9.9	0.7802	0.1406
Triglycerides (mg/dL)	100.8 ± 25.7	101.8 ± 33.2	117.3 ± 67.4	0.0169	0.0004
Glucose (mg/dL)	85.4 ± 10.0	92.2 ± 7.6	99.8 ± 16.5	0.0264	0.0173
Insulin (UI/mL)	6.4 ± 1.7	8.45 ± 4.2	23.1 ± 6.2	<0.0001	<0.0001
Alkaline phosphatase (UI/L)	93 ± 45.2	112.9 ± 40.4	125.8 ± 47	0.3044	0.0923
Total bilirubin (mg/dL)	0.6 ± 0.2	0.7 ± 0.2	0.7 ± 0.3	0.1883	0.9803
Direct bilirubin (mg/dL)	0.2 ± 0.01	0.2 ± 0.04	0.2 ± 0.1	0.0524	0.5823
HOMA-IR	1.08 ± 0.22	1.08 ± 1.1	5.6 ± 2.2	<0.0001	<0.0001
SBP (mmHg)	133 ± 9.8	135 ± 8.9	140 ± 11.7	0.2268	0.0684
DBP (mmHg)	70.4 ± 2.9	73.4 ± 5.2	82.2 ± 8.8	0.0072	< 0.0001
Leucocyte (10^3^/*μ*L)	4.66 ± 0.9	5.12 ± 0.8	6.6 ± 2.0	0.0359	0.0586
Neutrophil (10^3^/*μ*L)	1.86 ± 0.13	1.9 ± 0.4	4.1 ± 1.3	0.0002	0.0003
Lymphocyte (10^3^/*μ*L)	1.65 ± 0.31	1.6 ± 0.4	1.9 ± 0.6	0.6981	0.3611
NLR	1.1 ± 0.27	1.3 ± 0.5	2.4 ± 0.8	0.0021	0.0093

BMI: body mass index; AST: aspartate aminotransferase; ALT: alanine aminotransferase; GGT: Gamma glutamil transferase; HOMA-IR: homeostasis model assessment of insulin resistance; LDL: Low Density Lipoprotein; HDL: High Density Lipoprotein; SBP: systolic blood pressure; DBP: systolic blood pressure; NLR: Neutrophil-to-lymphocyte ratio. Normally distributed date described as mean, standard deviations (SDs), *P* value, ^∗^*P* < 0.05, ^∗∗^*P* < 0.01, ^∗∗∗^*P* < 0.001, ^∗∗∗∗^*P* < 0.0001, and unpaired Mann-Whitney *U* test.

**Table 2:** Histological characteristics of NASH liver tissues.

Score	NASH patients *n* = 20
Steatosis	
0	
1	2 (10%)
2	6 (30%)
3	12 (60%)
Inflammation	
0	2 (10%)
1	5 (25%)
2	13 (65%)
Ballooning	
0	1 (5%)
1	7 (35%)
2	12(60%)
NAS	
5	10(50%)
6	4 (20%)
7	6 (30%)
Fibrosis	
0	6 (30%)
1	11(55%)
2	3 (15%)
3	0

According to semiquantitatively score described by Gunn and Shiffman [34], the presence of NASH was defined according to the NAFLD activity score (NAS) and the histological features of liver biopsies scored as reported above. The data are showed as prevalence case N (%).

**Table 3:** Fluorochrome-antibody combinations used for flow cytometry.

Human	Fluorochrome	Clone	Company	Dilution in 100 *μ*L staining volume	Catalogue number and RRID
CD62L	FITC	DREG-56	BioLegend	1 : 100 (1 *μ*L)	Cat# 304803, RRID:AB_314463
CD16	PE	3G8	BioLegend	1 : 100 (1 *μ*L)	Cat# 980102, RRID:AB_2616616
CD56	PE/dazzle	HCD56	BioLegend	1 : 40 (2.5 *μ*L)	Cat# 362543, RRID:AB_2565921
CD14	PerCP/Cy5.5	HCD14	BioLegend	1 : 50 (2 *μ*L)	Cat# 325621, RRID:AB_893252
CD3	PerCP/Cy5.5	OKT3	BioLegend	1 : 50 (2 *μ*L)	Cat# 344807, RRID:AB_10641704
CD11b	PE/Cy7	ICRF44	BioLegend	1 : 40 (2.5 *μ*L)	Cat# 301321, RRID:AB_830643
CD45	APC	2D1	BioLegend	1 : 100 (1 *μ*L)	Cat# 368511, RRID:AB_2566371
CD3	BrilliantViolet605	OKT3	BioLegend	1 : 70 (1.4 *μ*L)	Cat# 317331, RRID:AB_2561376
CD4	BrilliantViolet711	RPA-T4	BioLegend	1 : 70 (1.4 *μ*L)	Cat# 300536, RRID:AB_2632791
HLADR	BrilliantViolet711	L243	BioLegend	1 : 70 (1.4 *μ*L)	Cat# 301045, RRID:AB_11219195
CD19	BrilliantViolet785	HIB19	BioLegend	1 : 70 (1.4 *μ*L)	Cat# 302239, RRID:AB_11218596
CD8	Brilliant Violet785	RPA-T8	BioLegend	1 : 70 (1.4 *μ*L)	Cat# 301045, RRID:AB_11219195

## Data Availability

The data used to support the findings of this study are included within the article.

## Abbreviations

NAFLD: Nonalcoholic fatty liver disease
NAFL: Nonalcoholic fatty liver
NASH: Nonalcoholic steatohepatitis
PMNs: Polymorphonuclear leukocytes
MPO: Myeloperoxidase
ROS: Reactive oxygen species
ARG1: Arginase 1
PBMCs: Peripheral blood mononuclear cells
CFSE: Carboxyfluoresceindiacetate, succinimidyl ester
H2DCF-DA, 2′: 7′-dichlorodihydrofluorescein diacetate
MFI: Mean fluorescence intensity
PB: Peripheral blood
LPS: Lypopolysaccharide.

## Supplementary Materials

Supplementary Materials Supplementary Figure S1: representative flow cytometry gating strategy of PMNs of NASH patients after 24 hrs of coculture with PBMC. FACS plots displaying, CD16 versus SSC-A, CD11b versus CD62L in coculture PMN analysis. Supplementary Figure S2: fold change in the ROS production of PMNs and PMNs coculture with PBMCs. All data are represented as mean ± standard deviation (^∗^*P* < 0.05). Supplementary Figure S3: representative gating strategy of PMNs derived from NASH patients cocultured or not with PBMCs. The neutrophil population (PMNs) in peripheral blood was distinguished by flow cytometry based on their forward- (FSC-A) and side-scatter (SSC-A) characteristics. (A) PBMCs cultured without CD3/CD28 stimulation have been used as internal control. PBMCs obtained from NASH patients were stimulated with aCD3/aCD28 and cultured with (B) and without (C) autologous PMNs for 96 h. Capacity of PMNs to suppress T cells was evaluated by measuring proliferating T cells (CFSE dilution).
