# Supplementary material for: Circulating Neutrophils of Nonalcoholic Steatohepatitis Patients Show an Activated Phenotype and Suppress T Lymphocytes Activity
Source: J Immunol Res. 2020 Jun 30;2020:4570219. doi: 10.1155/2020/4570219 (PMC7346102; doi:10.1155/2020/4570219)
Supplement: Supplementary Materials — Supplementary Figure S1: representative flow cytometry gating strategy of PMNs of NASH patients after 24 hrs of coculture with PBMC. FACS plots displaying, CD16 versus SSC-A, CD11b versus CD62L in coculture PMN analysis. Supplementary Figure S2: fold change in the ROS production of PMNs and PMNs coculture with PBMCs. All data are represented as mean ± standard deviation (∗P < 0.05). Supplementary Figure S3: representative gating strategy of PMNs derived from NASH patients cocultured or not with PBMCs. The neutrophil population (PMNs) in peripheral blood was distinguished by flow cytometry based on their forward- (FSC-A) and side-scatter (SSC-A) characteristics. (A) PBMCs cultured without CD3/CD28 stimulation have been used as internal control. PBMCs obtained from NASH patients were stimulated with aCD3/aCD28 and cultured with (B) and without (C) autologous PMNs for 96 h. Capacity of PMNs to suppress T cells was evaluated by measuring proliferating T cells (CFSE dilution). [file 4570219.f1.pdf]

Supplementary Figure S1

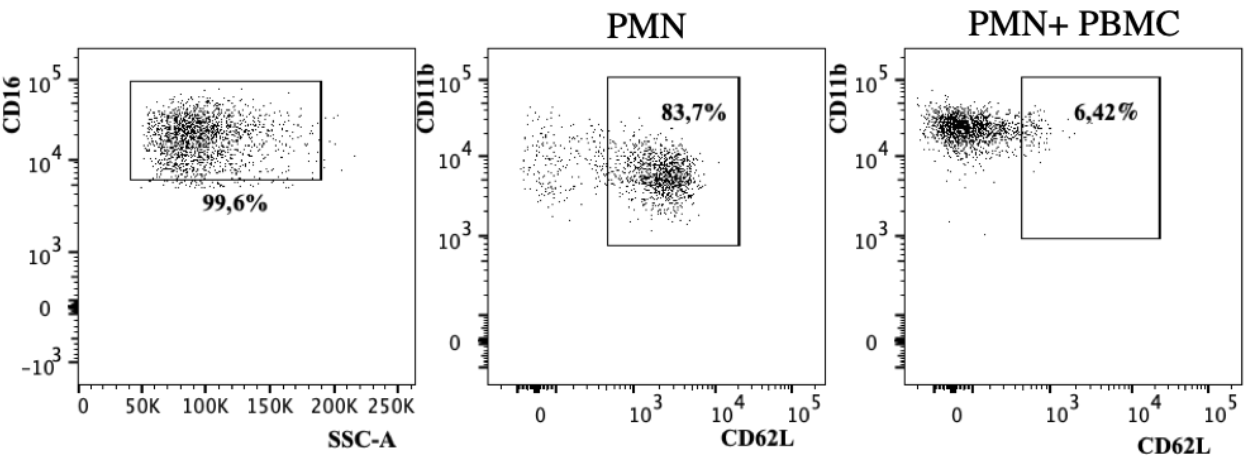

Figure S1. Representative flow cytometry gating strategy of PMNs of NASH patients after 24 hrs of co-culture with PBMC. FACS plots displaying, CD16 versus SSC-A, CD11b versus CD62L in co-culture PMNs analysis.

Supplementary Figure S2

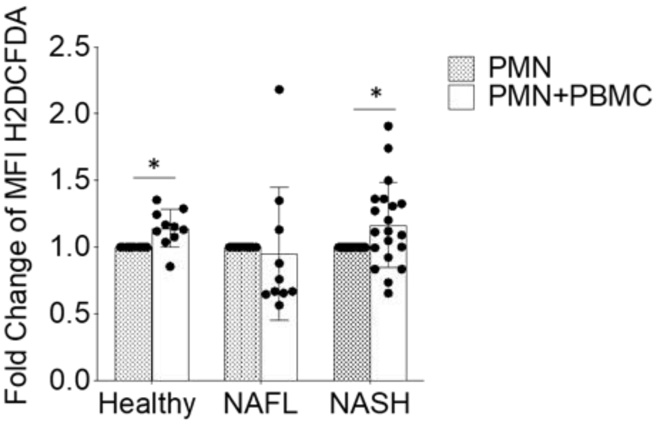

Figure S2. Fold change in ROS production of PMNs and PMNs co-culture with PBMCs. All data are represented as mean ± standard deviation (*P<0.05).

Supplementary Figure S3

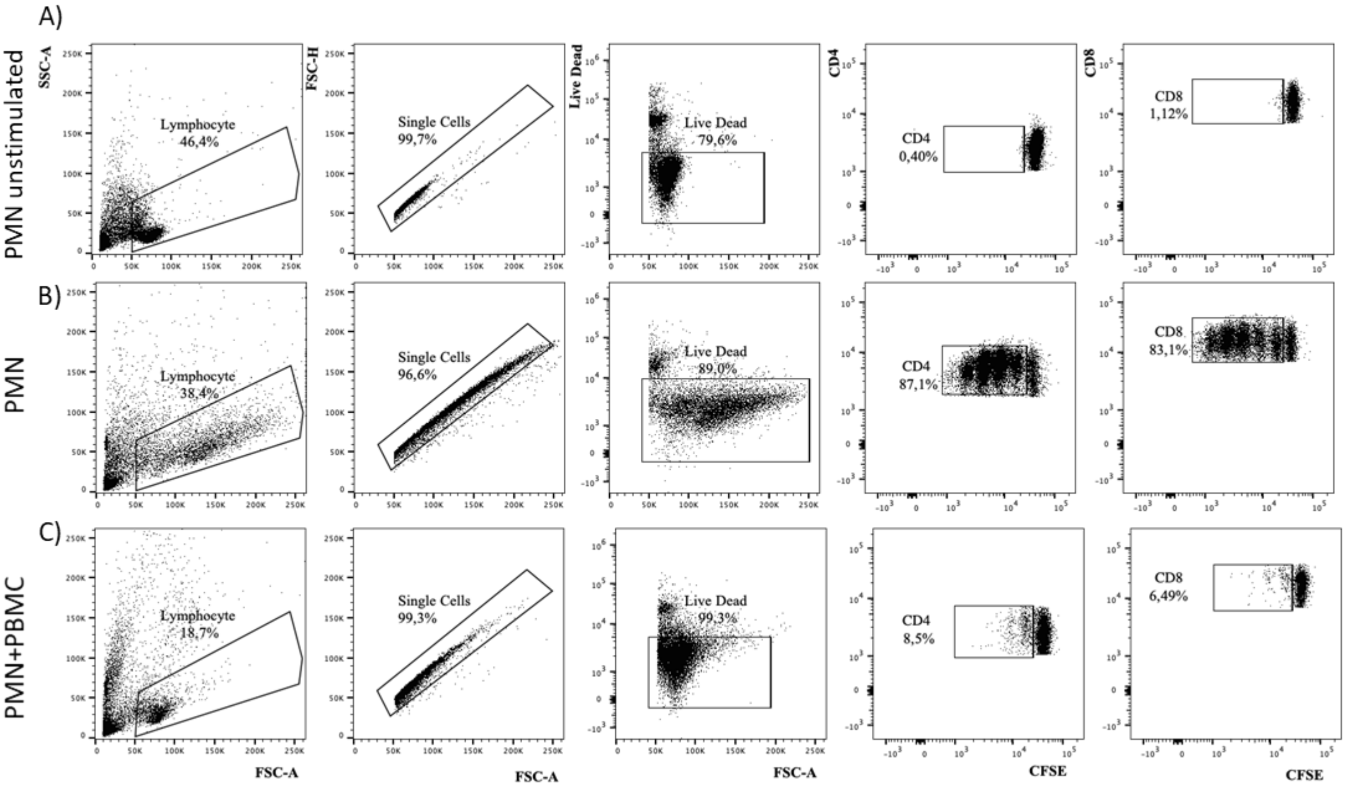

Figure S3. Representative gating strategy of PMNs derived from NASH patients co-cultured or not with PBMCs. The neutrophil population (PMNs) in peripheral blood was distinguished by flow cytometry based on their forward- (FSC-A) and side-scatter (SSC-A) characteristics. (A) PBMCs cultured without CD3/CD28 stimulation have been used as internal control. PBMCs obtained from NASH patients were stimulated with aCD3/aCD28 and cultured with (B) and without (C) autologous PMNs for 96h. Capacity of PMNs to suppress T cells was evaluated by measuring proliferating T cells (CFSE dilution).
